# Overexpression of p65 attenuates celecoxib-induced cell death in MDA-MB-231 human breast cancer cell line

**DOI:** 10.1186/1475-2867-13-14

**Published:** 2013-02-12

**Authors:** Ling Wang, Fubiao Kang, Jie Li, Jing Zhang, Baoen Shan

**Affiliations:** 1 Hebei Cancer Institute, the Fourth Hospital of Hebei Medical University, Shijiazhuang, Hebei, PR China; 2 Department of Liver Diseases, Bethune International Peace Hospital, Shijiazhuang, Hebei, PR China; 3 Medical department, the Fourth Hospital of Hebei Medical University, Shijiazhuang, Hebei, PR China; 4 Department of Information Management, the Fourth Hospital of Hebei Medical University, Shijiazhuang, Hebei, PR China; 5 Scientific Research Center, the Fourth Hospital of Hebei Medical University, Shijiazhuang, Hebei, PR China

**Keywords:** Breast cancer, p65, Celecoxib, Apoptosis

## Abstract

**Background:**

Celecoxib is a selective cyclooxygenase (COX)-2 inhibitor that has been reported to reduce the risk of breast cancer. In our previous study, celecoxib induced apoptosis and caused cell cycle arrest at the G0/G1 phase in the breast cancer cell line MDA-MB-231, and its effects were mediated by downregulation of NF-κB signaling. The NF-κB p65/RelA subunit may play a role in cell death through the activation of anti-apoptotic target genes including the inhibitor of apoptosis (IAP) and Bcl-2 families, and inhibition of protein kinase B/Akt. The aim of the present study was to investigate p65 as the potential target of celecoxib treatment and determine whether p65 overexpression can override the inhibitory effect of celecoxib on NF-κB activity and affect cell survival.

**Methods:**

The effects of p65 overexpression on celecoxib-inhibited NF-κB transcriptional activity were examined by western blotting, electrophoretic mobility shift assay (EMSA) and luciferase reporter gene assay. Cell viability and cell death were evaluated by the 3-(4,5-dimethylthiazol-2-yl)-2,5-diphenyltetrazoliumbromide (MTT) assay, and the levels of cleaved poly(ADP-ribose) polymerase (PARP) and caspase. Anti-apoptotic NF-κB target genes and cell cycle regulators were examined by western blotting to screen for the expression of target genes under direct regulation by p65.

**Results:**

Overexpression of p65 increased NF-κB transcriptional activity and interfered with celecoxib-mediated apoptosis as assessed by MTT assay and caspase-3, caspase-9, and PARP expressions. Exogenously overexpressed p65 upregulated NF-κB-responsive genes, including anti-apoptotic genes such as survivin and XIAP, and the cell cycle regulatory gene cyclin D1. However, p65 overexpression did not affect celecoxib-induced p-Akt inactivation, suggesting that celecoxib might have separate molecular mechanisms for regulating Akt signaling independently of its inhibition of NF-κB transcriptional activity.

**Conclusions:**

p65 is a pivotal anti-apoptotic factor that can reverse celecoxib-induced growth inhibition in MDA-MB-231 cells.

## Background

Breast cancer is currently the most common form of cancer affecting women and the sixth leading cause of cancer-related death among women in China [1]. Despite intensive cancer control efforts, its treatment remains a significant challenge for physicians [2]. Celecoxib is a selective cyclooxygenase (COX)-2 inhibitor that has been widely marketed as an anti-inflammatory drug with improved safety and lower toxicity than other nonsteroidal anti-inflammatory drugs (NSAIDs). It exerts potent anticancer effects in various tumor types, including colon, skin, prostate, lung and breast cancers [3-8]. The anti-tumorigenic effects of celecoxib are mediated by cell growth inhibition, and induction of apoptosis and cell cycle arrest. However, the exact molecular mechanisms involved are not yet well defined. Recent studies have identified new molecular targets for celecoxib that are associated with several signaling pathways including FLICE-inhibitory protein (c-FLIP)/glycogen synthase kinase-3 (GSK3) [9], Wnt/β-catenin [10], IL-6/JAK2/STAT3 [11], nuclear factor-kappa B (NF-κB) [12] and Akt [13].

The transcriptional factor NF-κB plays pivotal roles in several cellular pathways and has received increasing attention in recent years. The activation of NF-κB signaling has been reported in breast cancer cell lines and tumors [14,15]. The NF-κB family is composed of five Rel-domain-containing proteins, p65/RelA, RelB, c-rel, p50/NF-κB1 and p52/NF-κB2. In most cells, the predominant form of active NF-κB consists of a p65/p50 heterodimer that is retained in the cytoplasm in an inactive form, and is bound by an inhibitory protein called inhibitor of κB (IκB) [16,17]. The p65 subunit of NF-κB is responsible for the transactivation of downstream genes involved in the regulation of cell survival, cell cycle distribution, and apoptosis [18]. In particular, p65 activation is a predictive factor of resistance to neoadjuvant chemotherapy in breast cancer patients [19]. However, the specific role of p65 in breast cancer is still unclear.

In a previous study, Basu et al. [13] reported that celecoxib inhibited the growth of MDA-MB-231 human breast cancer cells through the inactivation of protein kinase B/Akt. In addition, work from our group showed that celecoxib could downregulate p65 level and the transactivation activity of NF-κB, which promoted cell apoptosis through the activation of the proapoptotic proteins PARP and caspase-3 in MDA-MB-231 cells [20]. However, we are not sure whether p65 is the direct target of celecoxib. Moreover, the relationship between Akt and NF-κB is controversial and elusive [21,22]. To further our understanding of the effect of p65 in MDA-MB-231 cells, p65 was overexpressed and its role in counteracting celecoxib-induced apoptosis and cell cycle arrest was evaluated. The results of our study may provide insight into the mechanism of action of celecoxib and help elucidate the role of p65 in breast cancer cells.

## Materials and methods

### Reagents

Celecoxib with a purity of 98% was purchased from Pharmacia (Skokie, IL) and dissolved in DMSO to generate a 100 mM stock solution that was stored at −20°C. Penicillin, streptomycin, Dulbecco’s modified eagle’s medium (DMEM), fetal bovine serum (FBS), and Lipofectamine 2000 were obtained from Invitrogen Life Technologies (Invitrogen, USA). The NE-PER nuclear and cytoplasmic extraction reagent kit and lightshift chemiluminescent electrophoretic mobility shift assay (EMSA) kit were purchased from Pierce Biotechnology (Pierce Biotechnology, Rockford, IL). The cell death detection ELISA^PLUS^ assay was purchased from Roche (Roche Diagnostics Corp., Indianapolis, IN). All other reagents were obtained from Sigma (St. Louis, MO). All antibodies were from Santa Cruz Biotechnology (Santa Cruz, CA).

### Cell culture

MDA-MB-231 cells were obtained from the American Type Culture Collection (ATCC) and routinely cultured in DMEM containing 10% FBS in the presence of 100 U/ml penicillin and 100 μg/ml streptomycin. Cells were incubated at 37°C with 95% air and 5% carbon dioxide. All cells were used in experiments during the linear phase of growth.

### Plasmids

The p65 expression vector (pcDNA3.1-p65) was a kind gift from Dr Bu youquan, Chongqing Medical University. The empty vector pcDNA3.1 was purchased from Invitrogen Life Technologies (Invitrogen, USA). The NF-κB-responsive luciferase reporter construct (NF- κB-p65-Luc), and its control plasmid (pRL-TK) were purchased from Promega (Promega,USA).

### Transient transfection and luciferase assay

MDA-MB-231 cells were seeded at a concentration of 5 × 10^5^ cells/well in 24-well plates. After overnight culture and reaching 80% confluence, the cells in each well were transiently transfected with 1 μg DNA consisting of NF-κB-p65-Luc and pRL-TK in serum-free medium according to the manufacturer’s protocol. After 12 h, the transfection mix was removed and replaced with complete medium. Twenty-four hours after co-transfection, cells were harvested and luciferase activity was determined using Luciferase Assay System (Promega, E1500).

### Cell viability assay

MDA-MB-231 cells were seeded at a density of 5 × 10^3^ cells per well in 200 μl of DMEM medium into 96-well plates, and cultured overnight. Cells were treated with 80 μM celecoxib and incubated for 24, 48, and 72 h. At different time points, the medium was replaced with 100 μl fresh medium containing 0.5 mg/ml 3-(4,5-dimethylthiazol-2-yl)-2,5-diphenyl tetrasodium bromide (MTT). Four hours after the addition of MTT, the supernatants were removed and discarded, and 150 μl of DMSO was added to each well to dissolve the crystals. Cell viability was determined with a microplate reader at a wavelength of 490 nm. All plates had control wells containing medium without cells to obtain a value for background luminescence, which was subtracted from the test sample readings. Each experiment was performed in triplicate and repeated at least three times.

### Flow cytometry for cell cycle analysis

Flow cytometric analyses were performed to determine the cell cycle distribution. MDA-MB-231 cells were treated with vehicle or 80 μM celecoxib for 24 h, and then harvested by trypsinization and fixed with 70% ethanol. Cells were stained for total DNA content with a solution containing 50 μg/ml propidium iodide (PI) and 100 μg/ml RNase I in PBS for 30 min at 37°C. Cell cycle distribution was then analyzed with the FACS Calibur Flow Cytometer (BD, USA). The proportions of cells in the G0/G1, S and G2/M phases were analyzed by FACS and a DNA software program. This experiment was repeated three times and the results were averaged.

### EMSA

The DNA binding activity of NF-κB was confirmed with a biotin-labeled oligonucleotide NF-κB probe (5’-AGTTGAGGGGACTTTCCCAGGC-3’). Cells were plated in 6-well plates and grown to 70% confluency. Cells were preincubated with celecoxib for 24 h, after which nuclear extracts (10 μg) were prepared according to the manufacturer’s protocol (Pierce, 20148). After incubation with 1 μg/μl of poly (deoxyinosinic-deoxycytidylic acid) in binding buffer for 30 min at 4°C, the nuclear extract mixed with probe was resolved on a 6% polyacrylamide gel at 100 V for 1 h, transferred to a nylon membrane, and subjected to UV crosslinking, and membranes were exposed to X-ray film to visualize the bands.

### Apoptosis ELISA

Cells were plated in 96-well plates, starved by serum deprivation and allowed to attach for 24 h. Cells were pretreated with celecoxib at a concentration of 80 μM for 24, 48, and 72 h. After treatment, non-adherent and adherent cells were collected and apoptosis was assessed using the Cell Death Detection ELISA^PLUS^ Assay according to the manufacturer's instructions. Measurements were made using an ELISA reader at 405 nm and the results were expressed as the ratio of the absorbance of the celecoxib-treated cells to the absorbance of untreated cells. Treatment with each concentration of celecoxib was repeated in 6 wells, and each treated sample was normalized to media controls.

### Western blot analysis

For analysis of protein expression, cells were washed twice with ice-cold phosphate buffered saline (PBS) and extracted using the NE-PER nuclear and cytoplasmic extraction kit according to the manufacturer’s instructions. Proteins (20 μg/lane) were separated by 10% SDS-PAGE and electrotransferred to nitrocellulose. Membranes were blocked for 1 h at room temperature in 5% milk. NF-κB p65, caspase-3, caspase-9, PARP, cyclinD1, cyclin E, CDK2, CDK4, CDK6, p21, p27, Bax, Bcl-2, Bcl-xl, XIAP, survivin and GAPDH were detected by incubating the transferred membrane overnight at 4°C with the indicated mouse polyclonal antibody (Santa Cruz, USA) at a 1:1000 dilution, then washing and incubating with the appropriate HRP-conjugated secondary antibodies (1:5000) for 1 h at room temperature. Protein bands were visualized using the enhanced chemiluminescence method. Relative protein levels were calculated by normalization to the amount of GAPDH protein. Data shown are representative of 3 independent experiments.

### Statistical analysis

Results were expressed as mean values ± SD. Differences between groups were compared by one-way ANOVA, and the Student’s *t* test was used to evaluate statistical significance using SPSS software. A value of less than 0.05 (*P* < 0.05) was considered statistically significant.

## Results

### Overexpression of p65 increases NF-κB transcriptional activity in MDA-MB-231 cells

MDA-MB-231 cells express constitutively active NF-κB, and its activity could be significantly suppressed by celecoxib in our preliminary study. To further examine the biological functions of p65, a recombinant plasmid expressing p65 (p65cDNA) was constructed and transfected into MDA-MB-231 cells. Western blotting results confirmed that p65 expression was strongly upregulated in p65-transfected cells even after celecoxib (80 μM) treatment. However, there were no significant differences in p50 and IκBα expression between control- and p65-transfected cells.

**Figure 1:**
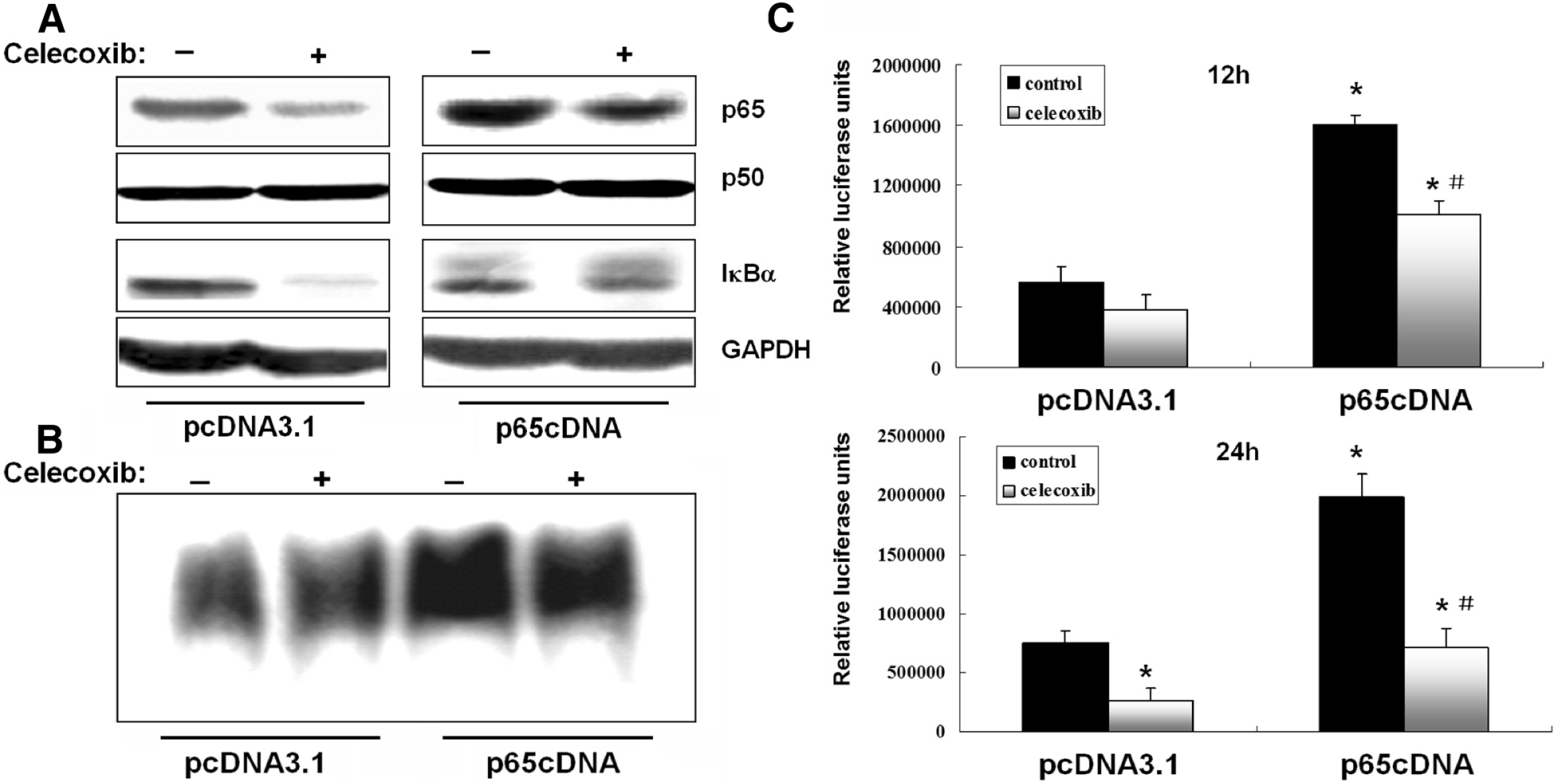
**Overexpression of p65 increases NF-κB transcriptional activity.** (**A**) Cells were transfected with p65cDNA or empty vector and treated with celecoxib (80 μM) for 12 h. (**B**) NF-κB DNA binding activity was evaluated by EMSA in cells transfected with p65cDNA or empty vector. (**C**) NF-κB DNA binding activity was evaluated by luciferase reporter gene assay in cells transfected with p65cDNA or empty vector. *P < 0.05, significantly different from empty vector transfected cells. #*P* < 0.05, significantly different from empty vector transfected cells after celecoxib treatment.

Overexpression of p65 caused a significant increase in NF-κB transcriptional activity as measured after 12 and 24 h by EMSA and luciferase reporter assays, respectively (Figure 1B and C). NF-κB transcriptional activity was only slightly affected by celecoxib pretreatment after 12 h in control- and p65-transfected cells, whereas significant inhibition was observed after 24 h. As expected, NF-κB transcriptional activity was significantly higher in p65-transfected cells even after celecoxib treatment.

### Overexpression of p65 does not affect Akt signaling

**Figure 2:**
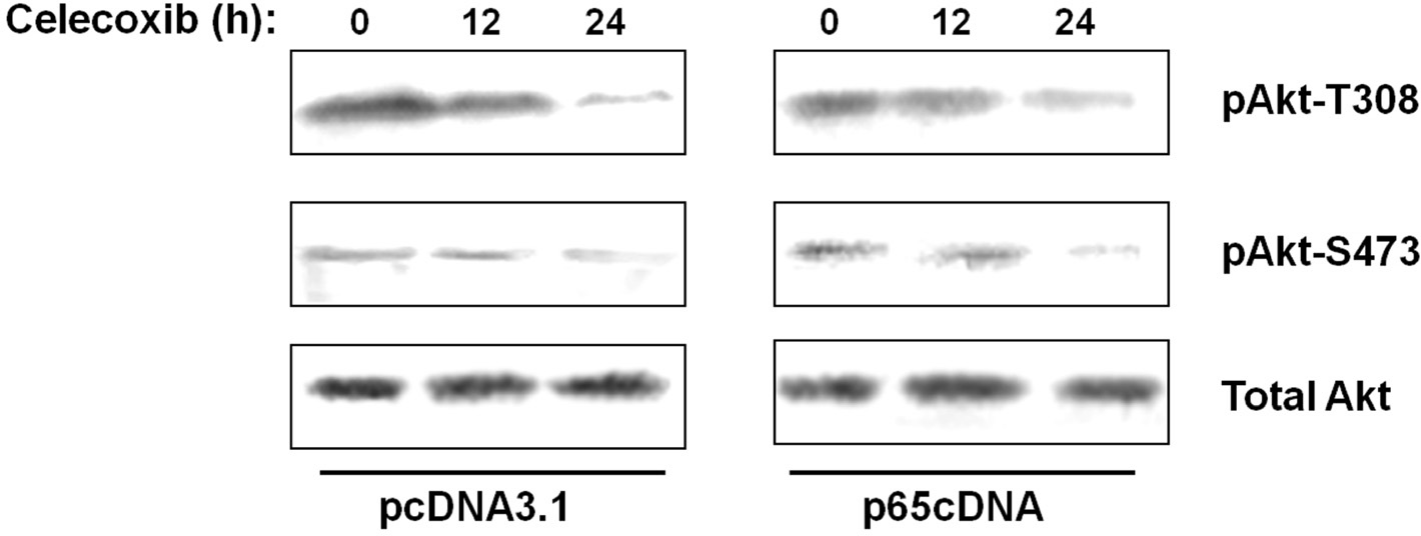
Inactivation of p-Akt by celecoxib is unaffected by overexpression of p65.

A study by Basu [13] and our previous study indicated that celecoxib induced apoptosis and cell cycle arrest of breast cancer cells by blocking Akt and NF-κB activation in vitro. The crosstalk between the Akt and NF-κB signaling pathways is not well understood. To determine the effects of p65 overexpression on Akt activation in MDA-MB-231 cells, cells were transiently transfected with p65cDNA and empty vector. As shown in Figure 2, celecoxib treatment decreased Akt phosphorylation at both S473 and T308. However, overexpression of p65 did not have a significant effect on the phosphorylation of Akt.

### p65 overexpression decreases celecoxib-induced apoptosis

To assess the effects of p65 overexpression on celecoxib-induced apoptosis, MDA-MB-231 cells were transfected with p65cDNA or empty vector and treated with 80 μM celecoxib for different time. The results of the MTT assay showed that treatment with 80 μM celecoxib for 24 h caused a loss of cell viability of 65.3%, which was reversed by p65cDNA transfection. These results indicated that p65 overexpression abrogated celecoxib-induced cell death.

**Figure 3:**
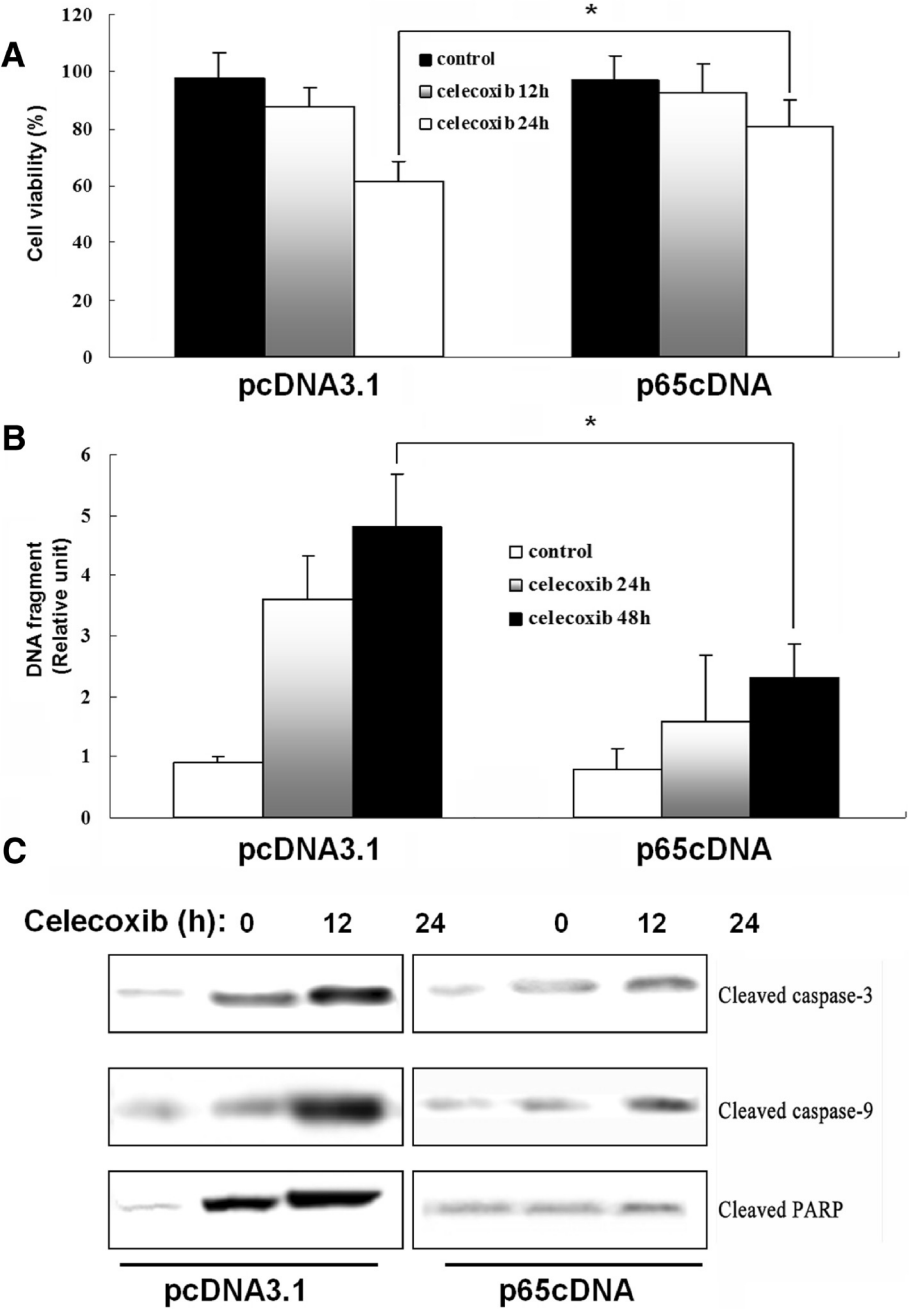
**p65 overexpression suppresses celecoxib-induced cell death.** (**A**) Cells were transfected with p65cDNA or empty vector and treated with celecoxib (80 μM) for 12 and 24 h. Cell death was assessed by MTT assay. (**B**) Cell apoptosis was assessed by Cell Death Detection ELISA^PLUS^ Assay. (**C**) Apoptosis was assessed by western blotting against cleaved PARP in whole-cell lysates.

The cell death detection ELISA^PLUS^ assay was used to assess the effect of p65 overexpression on celecoxib-induced cell death. The results indicated a 1.5-fold decrease in DNA fragmentation in p65-overexpressing cells compared with cells transfected with empty vector after treatment with celecoxib for 48 h. Caspases are responsible for many of the biochemical and morphological changes that occur during apoptosis [23]. In a previous study, we reported that activation of caspase-9, caspase-3 and PARP contributed to celecoxib-induced apoptosis in MDA-MB-231 cells. In the present study, p65-overexpressing cells had lower levels of cleaved caspase-9, caspase-3 and PARP than mock-transfected controls after celecoxib treatment (Figure 3C).

### p65 overexpression affects celecoxib downregulation of NF-κB-responsive anti-apoptotic genes

**Figure 4:**
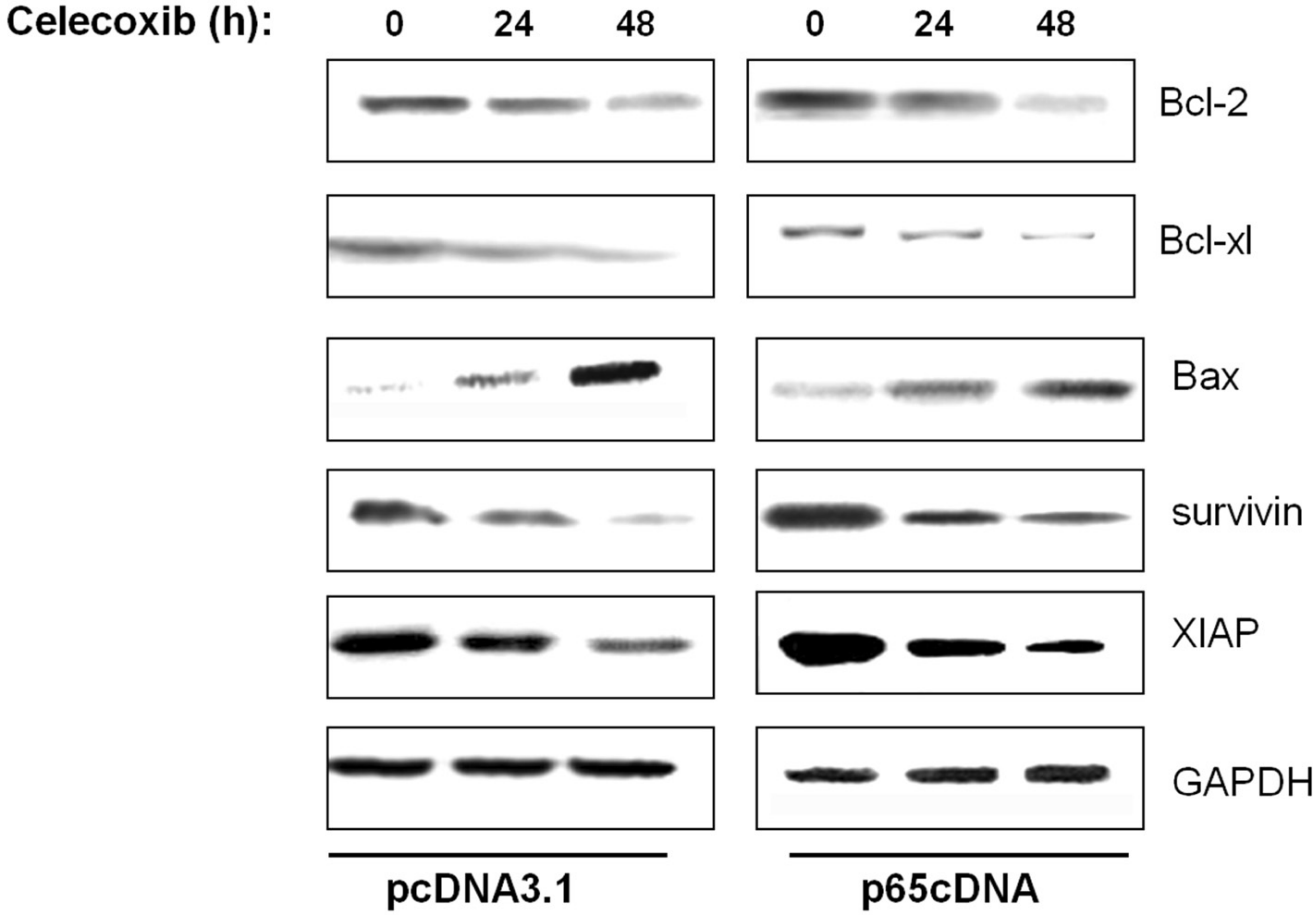
Effect of p65 overexpression on anti-apoptotic NF-κB target genes including Bcl-2 family and IAP family proteins.

The expressions of bcl-2, bcl-xL and bax were not significantly affected in control- and p65-overexpressing cells (Figure 4), but were reduced to similar degrees by celecoxib treatment in control and p65-overexpressing cells (Figure 4). These results indicated that the effect of celecoxib on the induction of apoptosis in MDA-MB-231 cells was mediated by bcl-2 family members, but not directly regulated by p65 expression, suggesting that p65 was not the only pivotal molecule regulating MDA-MB-231 cell survival.

The inhibitor of apoptosis proteins (IAPs) are a family of potent endogenous inhibitors that regulate apoptosis through direct inhibition of caspases or pro-caspases and are transcriptionally regulated by NF-κB [24]. Prior studies have shown that XIAP and suvivin are closely tied to the expression of the NF-κB p65 subunit [25,26]. As shown in Figure 4, XIAP and surivin expressions were significantly upregulated in p65-overexpressing cells compared to control cells. Celecoxib treatment reduced XIAP and surivin expression in mock-transfected cells but had no effect in p65-overexpressing cells at any time point.

### Celecoxib-induced G0/G1 phase cell cycle arrest is attenuated by p65 overexpression

Because changes in apoptosis alone could not completely account for the observed growth-inhibitory effects of celecoxib in MDA-MB-231 cells, cell cycle phase distribution was analyzed by flow cytometry. Following synchronization, p65cDNA-transfected cells and control cells were serum starved for 24 h and then treated with celecoxib for 24 h. Celecoxib treatment significantly increased the percentage of G0/G1 phase cells, and decreased the number of S and G2/M phase cells (Table 1). However, the celecoxib-induced increase in the number of G0/G1 phase cells was significantly reduced by p65 overexpression. Interestingly, basal cell cycle distribution in control cells was unaffected by p65 overexpression. These results indicated that p65 overexpression attenuated celecoxib-induced G1 phase arrest, but did not affect the cell cycle in wild MDA-MB-231 cells.

**Table 1:** p65 overexpression affects celecoxib-induced cell cycle arrest

**Group**	**G0/G1**	**G2/M**	**S**
pcDNA3.1	51.78 ± 5.09	19.81 ± 2.09	28.41 ± 2.07
p65cDNA	43.29 ± 4.12*	22.37 ± 1.76	34.34 ± 3.15*
pcDNA3.1 + 80μM celecoxib	62.07 ± 6.13	16.05 ± 1.98	21.88 ± 2.59
p65cDNA + 80μM celecoxib	50.98 ± 6.17^#^	20.31 ± 2.36^#^	28.71 ± 3.03^#^

*P < 0.05, pcDNA3.1vs p65cDNA;

^#^*P* < 0.05, pcDNA3.1 + 80 μM celecoxib vs p65cDNA + 80 μM celecoxib.

### The effect of celecoxib on cell cycle regulatory proteins is modulated by p65 overexpression

**Figure 5:**
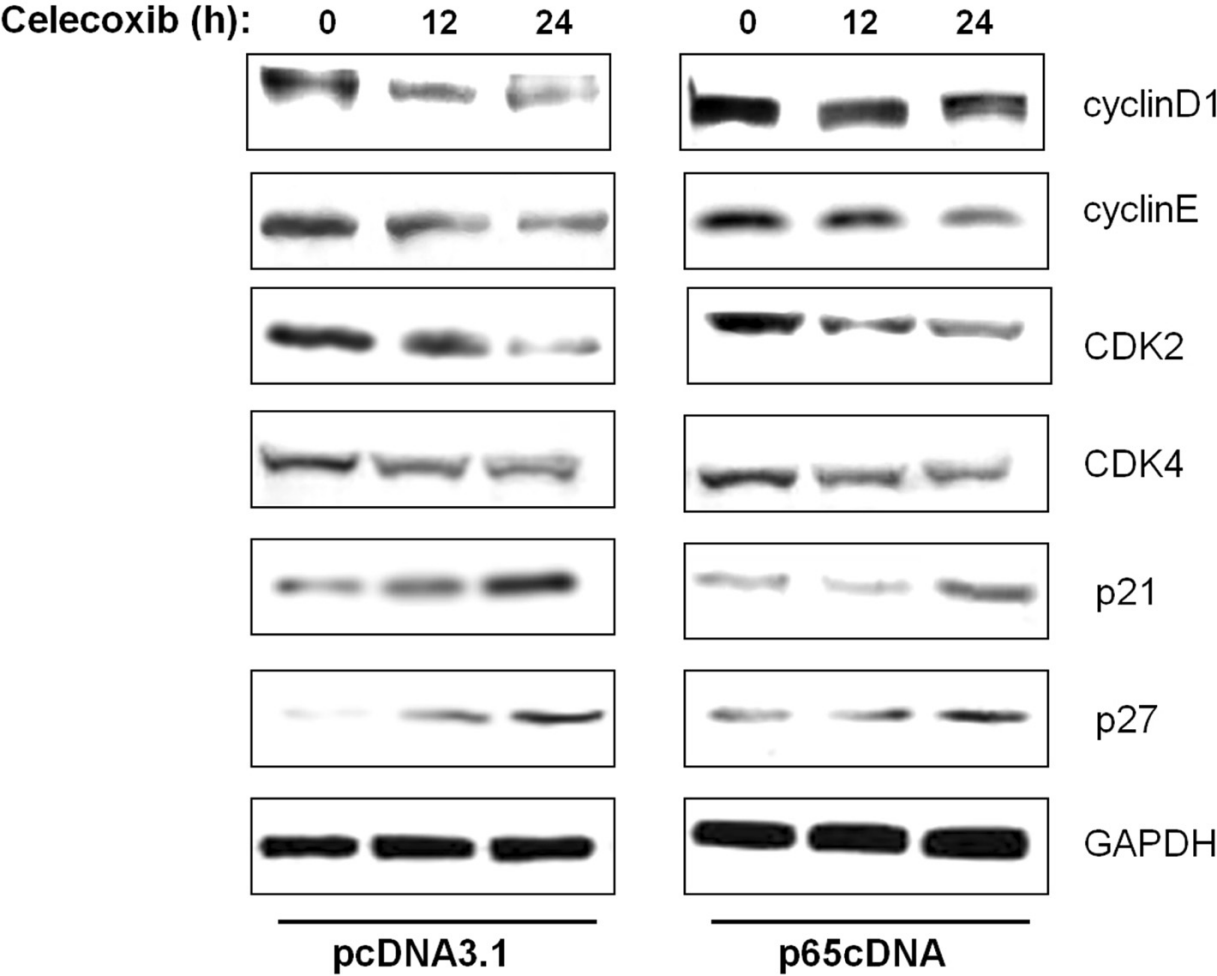
Examination of cell cycle regulators influenced by overexpression of p65.

To identify the potential contributors to the observed G_0_/G_1_ cycle arrest, cyclins, CDKs and CDKIs, which are associated with the G_1_/S checkpoint were analyzed by western blotting. As shown in Figure 5, cyclin D1, cyclin E, CDK2 and CDK4 were downregulated in a similar manner by celecoxib treatment. Compared with cells transfected with empty vector, p65-overexpressing cells showed high levels of cyclin D1 after celecoxib treatment. In contrast, no appreciable differences in the levels of cyclin E, CDK2 and CDK4 were observed between cells transfected with p65cDNA and empty vector. Among the CDKIs investigated, the expressions of p21 and p27 were upregulated in response to celecoxib treatment. p65 overexpression abrogated the celecoxib-induced p21 increment, but had no effect on p27.

## Discussion

We have shown previously that celecoxib could inhibit COX-2 and p65 expression levels, which promotes apoptosis in human MDA-MB-231 breast cancer cells [20]. Until now, the pro-apopotic effects of celecoxib have been proved to be associated with multiple intracellular signaling pathways including NF-κB, Akt and caspases, which interact to regulate programmed cell death [27]. To better understand the NF-κB/Akt signaling interactions implicated in celecoxib-mediated apoptosis, the present study overexpressed p65, the major transactivating subunit of NF-κB, in MDA-MB-231 cells and evaluated effects on celecoxib-induced Akt activation, NF-κB transcriptional activity, apoptosis and cell cycle distribution. In this study, overexpression of p65 in MDA-MB-231 cells enhanced p65 protein expression and the basal NF-κB DNA binding activity. However, transfection of MDA-MB-231 cells with p65cDNA had no significant effect on the NF-κB p50 subunit and the inhibitory molecule IκB. Celecoxib treatment, at 80 μM, which optimally induces apoptosis in MDA-MB-231 cells [20], inhibited NF-κB transcriptional activity and expression of NF-κB target genes. Celecoxib suppression of NF-κB was blocked in p65 transfectants since activity remained at least 2-fold higher than in mock-transfected cells. These results suggest that the exogenous upregulation of p65 could attenuate the inactivation of NF-κB caused by celecoxib treatment.

Akt has been shown to regulate cell survival and suppress apoptosis by stimulating the transactivation potential of the NF-κB p65 subunit [28]. In contrast, the NF-κB signaling pathway has also been reported to function upstream of Akt. Overexpression of p65 led to Akt phosphorylation in the absence of extracellular stimulatory factors and caused an increase in the expression of Akt at the mRNA and protein levels [21]. In MDA-MB-231 cells, celecoxib could inactivate pAkt with increased activation of proapoptotic protein [13]. In our previous study, we also found that celecoxib could inhibit cell growth, induce apoptosis and alter cell cycle distribution by blocking NF-κB signaling [20]. However, the present results indicated that overexpression of p65 had no effect on the phosphorylation of Akt, suggesting that Akt might play a role in upstream signaling pathways or via NF-κB-independent pathway in MDA-MB-231 cells. The precise molecular mechanisms remain to be determined.

NF-κB p65 can be an activator and repressor of its target genes depending upon the manner in which it is induced [29]. For example, Anto et al. [30] reported that overexpression of p65 in L929 mouse fibrosarcoma cells caused resistance to curcumin-induced apoptosis. Opposite results were reported by Collett et al. [31], who showed that overexpression of p65 potentiated curcumin-induced apoptosis in HCT116 human colon cancer cells. Besides, the role of p65 in the carcinogenic process is also complex and may involve the interaction of multiple signaling pathways in a context-specific manner. Yu et al. [32] reported that increased expression of p65 was correlated with colorectal tumorigenesis and promoted tumor progression. Conversely, Ricca et al. [33] reported that overexpression of p65 in MCF7/ADR cells reduced their tumorigenic ability in nude mice. The present study showed that overexpression of p65 could antagonize celecoxib-mediated apoptosis, as assessed by MTT assay and cell death fragment detection. Furthermore, transfected p65cDNA resulted in inhibition of caspase-3, -9 and PARP cleavage in MDA-MB-231 cells, which may be attributed to suppression of NF-κB-dependent transcriptional activity and expression of NF-κB-dependent anti-apoptotic genes. Therefore, we investigated two NF-κB-dependent target gene families, namely IAP family members and Bcl-2 family proteins. Results showed that p65 upregulation counteracted the celecoxib-induced inhibition of survivin and XIAP, while exerted no effects on Bcl-2 family members. The IAP family was confirmed to be regulated by NF-κB signaling and closely tied to breast cancer development [34]. The current study indicates that survivin and XIAP could be directly regulated by NF-κB p65 subunit, thus functioning anti-apoptosis in breast cancer cells. Although Bcl-2 has NF-κB binding sites and can be regulated by p65 [35], the expression of Bcl-2 family members remained unaffected in both mock transfectants and p65-overexpressing cells before and after celecoxib treatment. It is speculated that a putative increase in Bcl-2 mRNA might be counteracted by micro-RNA-mediated translational arrest [36].

Cell proliferation is the result of a rapid shift from a quiescent state to the progression of the cell cycle [37]. Flow cytometric results showed that celecoxib induced MDA-MB-231 cell cycle arrest at the G0/G1 phase, and this effect was suppressed by p65cDNA transfection. Progress in the eukaryotic cell cycle is driven by protein kinase complexes consisting of cyclins and CDKs. CDK activity is regulated negatively by a group of proteins called CDK inhibitors, including the protein p21 and p27 [38]. In the present study, cyclin D1 was increased in p65-overexpressing cells even in the presence of celecoxib, and p65 overexpression caused the inhibition of p21, suggesting a possible protective role for p65 expression in celecoxib-mediated cell cycle arrest. Collectively, these results indicated that p65 may have a direct role in regulating cell cycle progression, facilitating the transition of cells from G1 to S phase.

In conclusion, the present results strongly suggest that p65 expression has a protective role against celecoxib-mediated cell death of human breast cancer. In light of such a scenario, targeting p65 subunit or transfection with anti-p65 intrabody may enhance the anti-tumor effect of celecoxib in breast cancer treatment. Given that all current clinical anti-cancer drugs have reported incidences of drug resistance, development of experimental therapeutic aimed at new proliferative targets are of increasing importance. Further investigation into the molecular mechanism of p65 action may offer a novel approach to interfere with NF-κB activity in the nuclear compartment for treating breast cancer as well as other tumors.

## Competing interests

The authors declare that they have no competing interests.

## Authors’ contributions

LW and FK carried out the experiments. BS directed the whole experiment and provided the valuable comments editorial review of the manuscript. JL and JZ drafted the manuscript, edited, reviewed and finalized the data. All authors read and approved the final manuscript.
